# Effectiveness of Malodor-Reducing Ostomy Pouch Additives: An Assessment of Odor Intensity, Hedonic Tone, and Odor Character

**DOI:** 10.7759/cureus.65172

**Published:** 2024-07-23

**Authors:** Andrew Meacham, Philip Gowans, Jack Bradley-Clarke, Anne Swearingen, Suzanne Lord

**Affiliations:** 1 Sensory Testing, Olfasense UK Ltd., Bristol, GBR; 2 Ostomy Care, Convatec Ltd., Deeside, GBR; 3 Ostomy Care, Convatec Ltd., Lexington, USA

**Keywords:** pouch additives, malodor, prevention and control, odorants, ostomy

## Abstract

Background

Ostomy pouch odor can have a negative impact on the quality of life of people living with a stoma. This study assessed the effectiveness of malodor-reducing ostomy pouch additives under simulated conditions.

Methodology

The following six commercially available products with different odor control technologies plus a control were assessed: soyethyl morpholinium ethosulphate, zinc ricinoleate (ZnR), ZnR with orange terpenes (ZnR-Orange), a proprietary copper-based deodorant, a proprietary ion mix deodorant, and a terpene blend (TB). Each was added to an ostomy pouch with skatole (a substitute for human fecal odor). Professional olfactometrists rated odors according to intensity, hedonic tone (pleasantness), and character.

Results

The TB and ZnR-Orange had very weak (<1.0) malodor intensity, with mean (standard deviation [SD]) ratings of 0.6 (1.1) and 0.9 (0.9), respectively. All other products (2.7-3.0) and control (3.7) were statistically higher (stronger intensity) compared with the TB(p < 0.001). The mean (SD) hedonic tone for the TB was 0.8 (1.7) (considered slightly pleasant); all other products (-0.8 to 0.1) and control (-0.9) were statistically lower (p < 0.001). Odor character profiles were broadly comparable, but products with scent additives (TB and ZnR-Orange) were predominantly associated with fragrances.

Conclusions

This information may help nurses and other healthcare providers when educating ostomates about their options. Other factors such as application mode and recommended dosage may also influence the choice of product. Future research on real-world populations (i.e., ostomates), as well as assessment of lubrication properties, is warranted.

## Introduction

Stomas are created surgically for the treatment of conditions such as colon cancer and inflammatory bowel disease [1,2]. During surgery, a segment of the bowel is exteriorized to the abdominal surface creating an exit where fecal matter can be collected outside of the body in an ostomy system [2,3]. The procedure can be life-saving or necessary to reduce symptoms and can help patients return to a healthy life [2].

Despite the clinical benefits of ostomy procedures, a variety of issues affect the quality of life in people with ostomies (ostomates), including effects on intimacy, changes to mental health, flatulence and/or odor, or stress, fear, and anxiety related to social and/or work situations [2,4-11]. The impact can also extend to partners of ostomates [5,9].

Odor is a common concern, and, in some cases, ostomates may even feel that it defines them [4,7,12]. In two separate studies of ostomates, following surgery for colorectal cancer, 46.0% of patients indicated concern over odor in the postoperative period [12], and this was still a concern for 41.0% of patients at five months post-diagnosis [7]. Odor is particularly problematic when there is excess gas production while changing the pouch or during emptying, which ostomates do periodically during their day [7,13].

Odor pleasantness or unpleasantness can be quantified by hedonic tone and is measured independently of odor character [14]. Hedonic tone assessments are subjective, in that personal experience and memories of odors are used as a reference [14]. However, odor assessors can be trained in the use of referencing scales, with average values reported across assessors [14]. Understanding the perception of odor pleasantness by hedonic tone assessment is essential for developing appropriate methods to control odor.

There are a variety of home and commercially available remedies to minimize ostomy pouch odor. These include nutritional instructions [15], reduction strategies such as filters that contain ingredients to collect molecules associated with odor [13,15-17], and the use of additives [15,18]. For example, in a randomized study, ostomates who added lavender essential oil to their ostomy pouch had significantly fewer odor-related problems compared with the control group, and their quality of life improved [18]. Commercially available products to address odor include those that focus on odor alone and those that address odor but also lubricate the pouch. These are particularly useful when gases within the pouch do not pass through the filter, i.e., during emptying and changing. To our knowledge, there is no data directly comparing the multiple commercially available products for this purpose. Thus, the objective of this study was to assess the effectiveness of malodor-reducing ostomy pouch additives in terms of odor intensity, hedonic tone, and odor character.

## Materials and methods

Study design

This study was designed to assess the effectiveness of ostomy pouch additives at minimizing malodor perception during emptying, under simulated use conditions, in single-use ostomy pouches. The study was conducted in December 2021 at a single site in the United Kingdom. Eleven professional olfactometrists were screened for olfactory acuity and trained in the use of the intensity scale. Six dual-action (odor plus lubricant) ostomy pouch products with different odor control technologies were assessed, including soyethyl morpholinium ethosulphate (Hollister Adapt™ Lubricating Deodorant, Hollister Ltd., Wokingham, UK), zinc ricinoleate (ZnR; Be Free™ Lubricating Odor Eliminator, BeFree Technologies, Atlanta, GA, USA), ZnR with orange terpenes (ZnR-Orange; Coloplast Brava® Lubricating Deodorant, Coloplast Ltd., Peterborough, UK), a proprietary copper-based deodorant (Panasonic® Nioff Deodorant Lubricant, Panasonic Chemical Co., Ltd., Osaka, Japan), a proprietary ion mix deodorant (Safe n Simple™ Assure C Odor Eliminator, Safe n' Simple, Clarkston, MI, USA), and a terpene blend (TB; ESENTA™ Lubricating Deodorant, Convatec Ltd., Deeside, UK). A seventh pouch with no additional odor-reducing additives was included as a control. A skatole (3-methylindole) solution was used in each pouch as a surrogate for human fecal odor. Skatole is synthesized in the digestive tract of mammals and is considered a contributor to the smell of fecal matter [19]. The concentration of skatole can easily be controlled under laboratory conditions, promoting repeatability and reproducibility. Five replicates of each of the seven pouches were prepared, and each replicate was evaluated by a minimum of six olfactometrists for odor intensity, hedonic tone, and odor character.

Ostomy pouch preparation

Before testing, a stock solution of skatole was prepared in ethanol (50 mg in 40 mL) and diluted with de-ionized water to a final volume of 250 mL. The final dilution added to all products was 0.5 mL of the master stock solution diluted in water to make 100 mL (final skatole concentration of 7.62 µM). The target was to create a strong odor of intensity 4, which was determined by iterative dosing in a control test.

**Figure 1:**
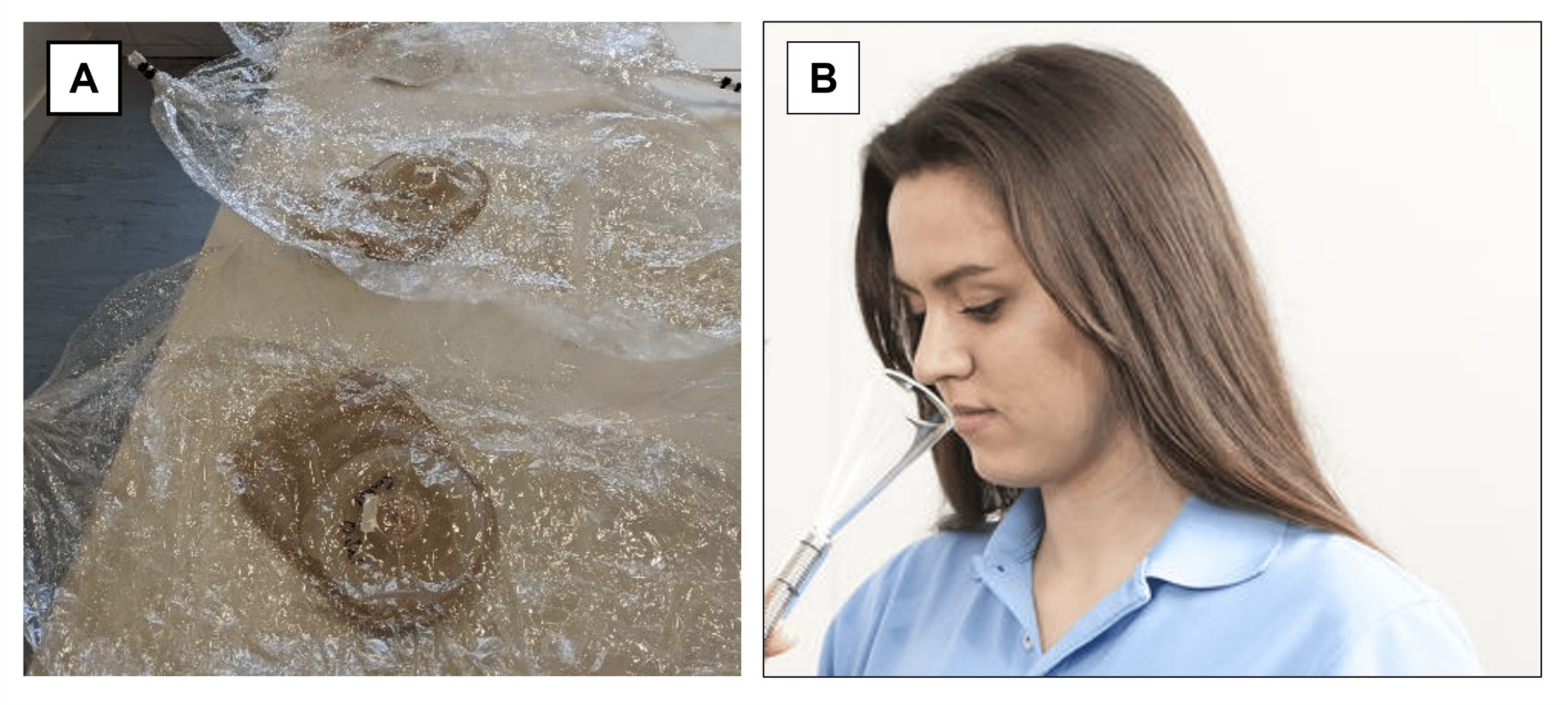
Ostomy pouches containing test samples inside gas sampling bags (A) and nosecone used to assess samples (B).

The six dual-action products and the control (water only) were each added, either as a spray (the TB) or liquid/drops (all other products) to a convex ostomy pouch (ESTEEM+™ Flex Convex, Convatec Ltd., Deeside, UK), according to the manufacturer’s dosing instructions, and gently massaged to ensure distribution. The water/skatole mixture was then added and the ostomy pouch opening was sealed. The pouches were placed into sealed sample enclosures (Nalophan®, Kalle GmbH, Wiesbaden, Germany) containing 20 L of clean air (Figure 1A). The sampling enclosures were left at room temperature for two hours and agitated gently every 30 minutes for 30 seconds to stimulate pouch movement. After two hours, the pouch seal was opened, and the liquid content was removed. The sampling enclosures were kept sealed to capture all emissions during pouch emptying. Following emptying, a further 20 L of clean air was added to the headspace of the original sampling enclosure and then transferred to a clean sample presentation bag before sensory analysis by olfactometrists. Samples were assessed by releasing air from the sample presentation bag via a nosecone (a funnel that surrounds the assessor’s nostrils, ensuring even distribution of the odor; Figure 1B). The testing process was repeated five times over three calendar days (five replicates for each of the seven pouches). Samples presented to the olfactometrists were visually indistinct and randomized.

Assessments

**Figure 2:**
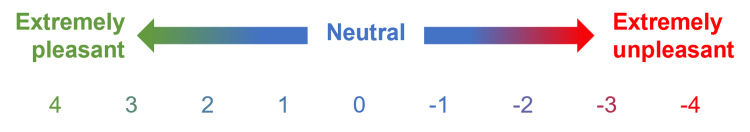
Hedonic tone scale. The scale ranges from 4 to -4, where 4 represents an extremely pleasant smell and -4 represents an extremely unpleasant smell.

Olfactometrists rated odors for the seven samples according to intensity, hedonic tone, and character. Intensity, the perceived strength of the skatole malodor, was ranked on a scale of 0 to 6, where 0 is non-detectable, and 6 is an extremely strong odor, based on the VDI reference scale 3882 Part 1 [20]. Hedonic tone, the relative pleasantness/unpleasantness of the odor, was ranked on a scale of -4 to +4, where -4 is considered extremely unpleasant and +4 extremely pleasant, based on the VDI reference scale 3882 Part 2 (Figure 2) [21]. Olfactometrists were asked to rank the overall pleasantness of the air sample assessed, including any fragrances present. Odor character, which provides descriptors to define odor categories, was measured by assigning descriptors to the perceived odors, based on a proprietary odor wheel from Olfasense. Olfactometrists were instructed to assign a primary scent category and any secondary categories as appropriate to the sample from the odor wheel.

Ethical considerations

Institutional review board approval was not required for this study as it was conducted by a panel of olfactory experts as part of their employment.

Statistical analysis

Odor intensity and hedonic tone data collected for each product were checked for normality using the Shapiro-Wilk test at a 5% significance level. The data tested were not normally distributed, and the non-parametric Wilcoxon signed-rank test was used to compare products. The hypotheses were to compare the TB and the other products to investigate whether there is a statistically valid difference between them. A 5% significance level was applied.

H0: The two samples follow the same distribution.

Ha: The distributions of the two samples are different.

Statistical tests were performed using XLSTAT Version 21.5.1, a statistical and data analysis solution (2021).

## Results

In total, 37 responses were obtained for each product or control, across five testing sessions/sets of samples and 11 olfactometrists.

Malodor intensity

**Figure 3:**
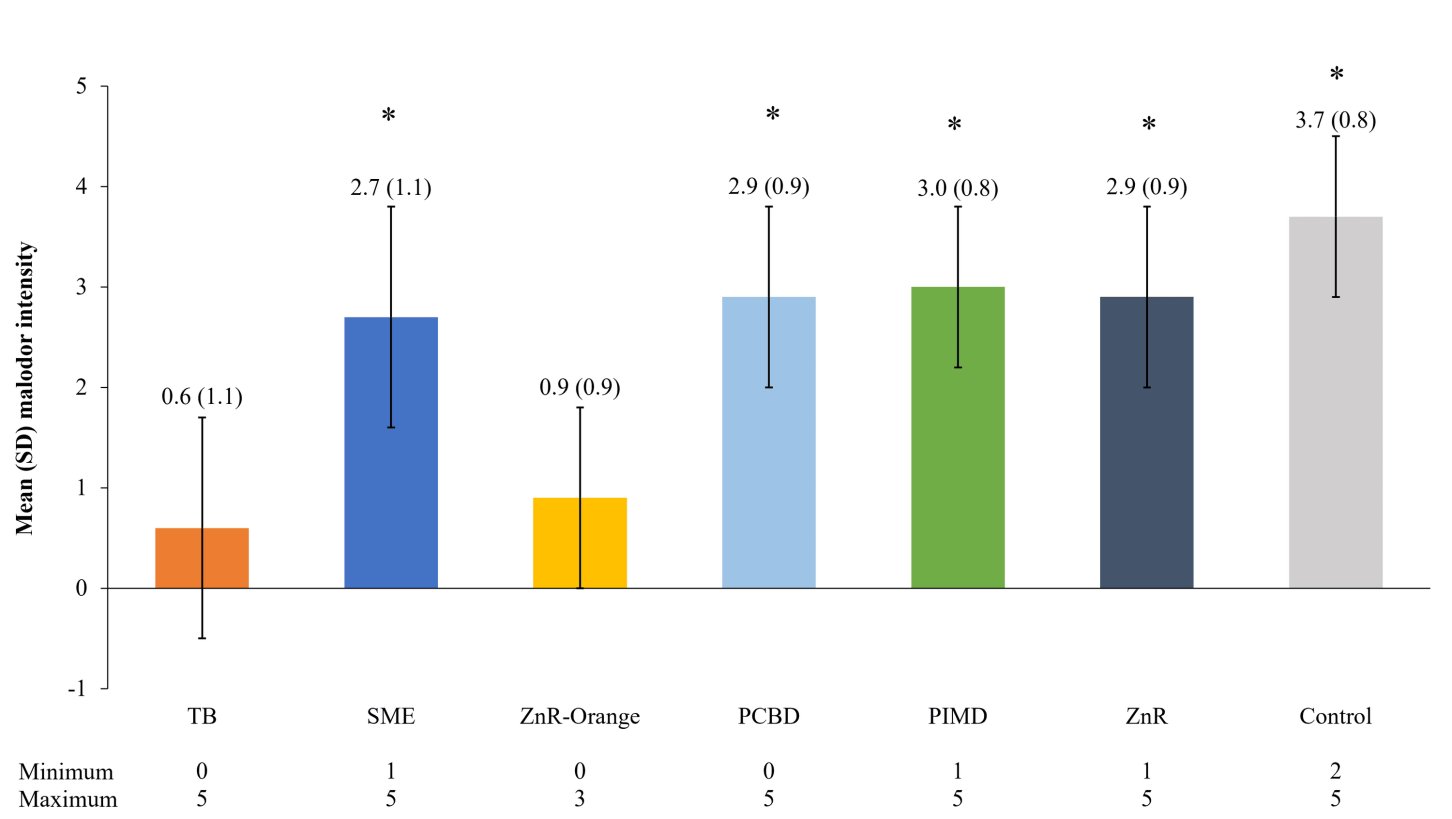
Perceived malodor intensity of ostomy pouch additives. *: p < 0.05 versus the TB (Wilcoxon signed-rank test). Five samples of each additive were ranked by at least six assessors each (37 observations per additive). Sample odors were ranked on an intensity scale of 0 to 6, where 0 is non-detectable and 6 is extremely strong. PCBD: proprietary copper-based deodorant; PIMD: proprietary ion mix deodorant; SD: standard deviation; SME: soyethyl morpholinium ethosulphate; TB: terpene blend; ZnR: zinc ricinoleate; ZnR-Orange: zinc ricinoleate with orange terpenes

The control pouch sample (untreated) resulted in the highest perception of malodor, with a mean (standard deviation [SD]) perceived odor intensity rating of 3.7 (0.8) (Figure 3). Scented products, the TB and ZnR-Orange, were rated as having a very weak (<1.0) malodor with mean (SD) ratings of 0.6 (1.1) and 0.9 (0.9), respectively. The mean perceived malodor intensity for the remaining products ranged from 2.7 to 3.0. Ratings were statistically higher (p < 0.05) for control and all other pouch additives compared with the TB, except for ZnR-Orange.

Hedonic tone

**Figure 4:**
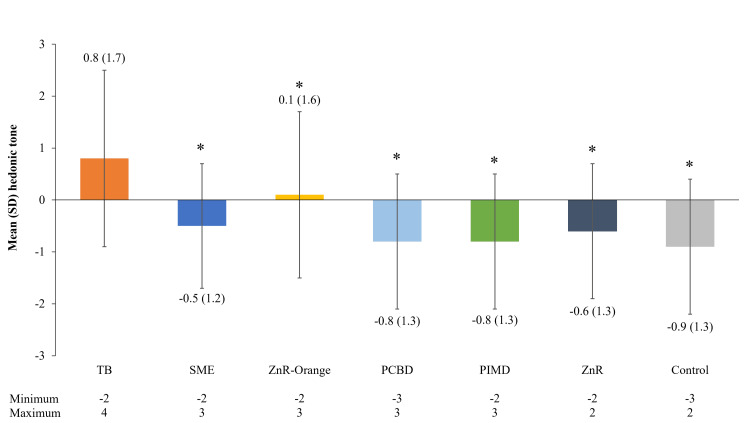
Hedonic tone of ostomy pouch additives. *: p < 0.05 versus the TB (Wilcoxon signed-rank test). Five samples of each additive were ranked by at least six assessors each (37 observations per additive). Sample odors were ranked for hedonic tone on a nine-point scale, where 4 is extremely pleasant, 0 is neither pleasant nor unpleasant, and -4 is extremely unpleasant. PCBD: proprietary copper-based deodorant; PIMD: proprietary ion mix deodorant; SD: standard deviation; SME: soyethyl morpholinium ethosulphate; TB: terpene blend; ZnR: zinc ricinoleate; ZnR-Orange: zinc ricinoleate with orange terpenes

The mean (SD) hedonic tone rating for the TB was 0.8 (1.7) (considered slightly pleasant; Figure 4). Only one other product (ZnR-Orange) was rated as neutral/slightly pleasant with a mean (SD) rating of 0.1 (1.6). The control sample and all other pouch additives generated negative or unpleasant hedonic tone scores, with mean ratings of -0.9 to -0.5 (slightly unpleasant to neutral). Ratings were statistically lower (p < 0.05) for control and all pouch additives compared with the TB.

Odor character

**Figure 5:**
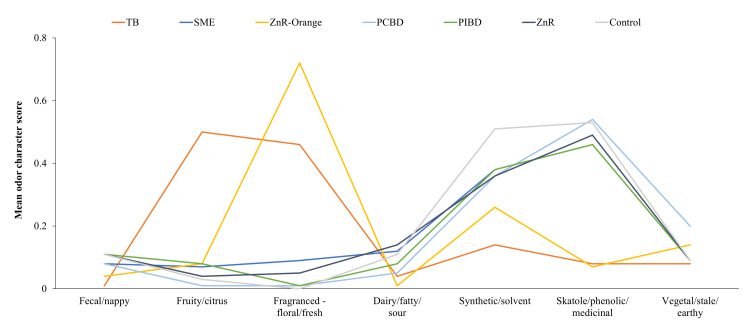
Odor character of ostomy pouch additives. Five samples of each additive were ranked by at least six assessors each (37 observations per additive). Sample odors were measured by assigning descriptors to the perceived odors, based on a proprietary odor wheel from Olfasense. PCBD: proprietary copper-based deodorant; PIMD: proprietary ion mix deodorant; SME: soyethyl morpholinium ethosulphate; TB: terpene blend; ZnR: zinc ricinoleate; ZnR-Orange: zinc ricinoleate with orange terpenes

Olfactometrists determined that most samples were broadly comparable to the control skatole (used in this study as a single component human fecal odor substitute) regarding odor character (Figure 5). Two products, the TB and ZnR-Orange, had odor character profiles that were predominantly associated with fragrances (both contain scent additives). ZnR-Orange scored slightly higher on synthetic/solvent characteristics compared with the TB.

## Discussion

Management of ostomy pouch odor is an important topic for healthcare providers due to its significant impact on ostomates and their families in terms of quality of life. With the numerous commercially available products, determining which product to select may be difficult. We evaluated six commonly marketed dual-action (odor plus lubricant) ostomy pouch additives and provided insights into important characteristics for the assessment of odor by showing a relationship between a lower perception of malodor and a positive hedonic tone. While previous studies have evaluated the effect of lavender essential oil on ostomy pouch odors [18,22], to our knowledge, our work represents the first to compare the characteristics of different malodor-reducing ostomy pouch additives.

The results demonstrate a difference in the effectiveness of the test ostomy pouch additives when three characteristics of odor, i.e., intensity, hedonic tone, and character, were evaluated. Of the products tested, the TB slightly outperformed ZnR-Orange in reducing malodor intensity. When the pleasantness of the odors associated with a range of pouch deodorants was evaluated (hedonic tone), the TB was ranked as the most pleasant of the products tested. Lastly, when all products were assigned a scent from an odor wheel, the two products with scent additives, the TB and ZnR-Orange, were considered to have scent profiles differing from the untreated skatole control. The scented products were more fragranced and floral, and, in the case of the TB, also had a fruity/citrus element. However, ZnR-Orange had slightly higher scores for less desirable odor characters compared with the TB, such as synthetic and solvent. The differing scent profiles of scented versus unscented products were not unexpected. However, the products with scent additives and with the lowest perception of malodor (the TB and ZnR-Orange) also demonstrated a more positive hedonic tone (pleasantness). This information may be helpful for healthcare providers when educating ostomates on their options for odor control. Other attributes that may impact malodor-reducing ostomy pouch additive choice include application mode (e.g., spray with the TB vs. liquid drops with the other products) and recommended dosage (e.g., 0.5 mL per application with TB vs. 8 mL with ZnR-Orange).

Several limitations of our work should be highlighted. First, the method utilized professional olfactometrists instead of ostomates who are not representative of the target population. Similarly, skatole was used rather than human excrement and therefore did not reflect real-world conditions. In addition, the test products were used according to the manufacturers’ instructions, which may not necessarily reflect actual ostomate use. The mode of application also differed between products (i.e., spray with the TB vs. liquid/drops with all other products). Lastly, of the tested dual-action additives, it is important to note that the TB and ZnR-Orange have added scent within their products.

## Conclusions

Differences in malodor intensity, hedonic tone, and character between the test ostomy pouch additives were observed. The TB and ZnR-Orange reduced malodor intensity to the greatest extent, and the TB was ranked as the most pleasant smelling (hedonic tone). Lastly, the TB and ZnR-Orange were the only products perceived to have scent profiles differing from the untreated skatole control. Collectively, this information may help nurses and other healthcare providers when educating ostomates about their options. Other factors such as application mode and recommended dosage may also influence the choice of product. Future research on real-world populations (i.e., ostomates), as well as assessment of lubrication properties, is warranted.
